# The relationship between patient physiology, the systemic inflammatory response and survival in patients undergoing curative resection of colorectal cancer

**DOI:** 10.1038/sj.bjc.6605919

**Published:** 2010-09-28

**Authors:** C H Richards, E F Leitch, P G Horgan, J H Anderson, R F McKee, D C McMillan

**Affiliations:** 1 University Department of Surgery, Faculty of Medicine, University of Glasgow, Royal Infirmary, Glasgow G31 2ER, UK

**Keywords:** colorectal cancer, GPS, inflammation, physiology, POSSUM, survival

## Abstract

**Background:**

It is increasingly recognised that host-related factors may be important in determining cancer outcome. The aim was to examine the relationship between patient physiology, the systemic inflammatory response and survival after colorectal cancer resection.

**Methods:**

Patients undergoing potentially curative resection of colorectal cancer were identified from a prospectively maintained database. Patient physiology was assessed using the physiological and operative severity score for the enumeration of mortality and morbidity (POSSUM) criteria. The systemic inflammatory response was assessed using the modified Glasgow Prognostic Score (mGPS). Multivariate 5-year survival analysis was carried out with calculation of hazard ratios (HR).

**Results:**

A total of 320 patients were included. During follow-up (median 74 months), there were 136 deaths: 83 colorectal cancer related and 53 non-cancer related. Independent predictors of cancer-specific survival were age (HR: 1.46, *P*<0.01), Dukes stage (HR: 2.39, *P*<0.001), mGPS (HR: 1.78, *P*<0.001) and POSSUM physiology score (HR: 1.38, *P*=0.02). Predictors of overall survival were age (HR: 1.64, *P*<0.001), smoking (HR: 1.52, *P*=0.02), Dukes stage (HR: 1.64, *P*<0.001), mGPS (HR: 1.60, *P*<0.001) and POSSUM physiology score (HR: 1.27, *P*=0.03). A relationship between mGPS and POSSUM physiology score was also established (*P*<0.006).

**Conclusion:**

The POSSUM physiology score and the systemic inflammatory response are strongly associated and both are independent predictors of cancer specific and overall survival in patients undergoing potentially curative resection of colorectal cancer.

Colorectal cancer is the second most common cause of cancer death in Western Europe and North America. Each year, in the United Kingdom, there are approximately 35 000 new cases and the disease accounts for over 16 000 deaths (http://www.cancerresearchuk.org). Overall survival is poor; even in patients who undergo resection with a curative intent, only half will be alive at 5 years (McArdle and Hole, 2002; Verdecchia *et al*, 2008).

Following curative resection, the prognostic stratification and provision of adjuvant therapy has traditionally been guided by the pathological analysis of the tumour (Figueredo *et al*, 2008). It is increasingly recognised, however, that pathological stage is not the sole determinant of outcome. Host-related factors, in particular the systemic inflammatory response, have proven important in determining outcome. There is now a considerable body of evidence that markers of systemic inflammation (in particular, C-reactive protein, albumin and their combination in the modified Glasgow Prognostic Score (mGPS)) can predict cancer-specific survival in patients undergoing curative resection of colorectal cancer (Roxburgh and McMillan, 2010a). This effect appears independent of TNM stage and other high-risk pathological features (Ishizuka *et al*, 2007; Koike *et al*, 2008; Roxburgh *et al*, 2009).

The basis of the relationship between systemic inflammation and survival is not clear and it has yet to be established which host characteristics, if any, an elevated inflammatory response may represent. It is of interest that a systemic inflammatory response has been reported to predict cardiac events (Lloyd-Jones and Levy, 2003) and is associated with patient-related factors such as obesity (Ridker *et al*, 2003), diabetes (Dehghan *et al*, 2007) and smoking (Fröhlich *et al*, 2003). One hypothesis, therefore, is that a pre-operative systemic inflammatory response may reflect the existence of co-morbid disease (Roxburgh and McMillan, 2010b) or altered patient physiology. Indeed, several studies have reported elevated physiological and operative severity score for the enumeration of mortality and morbidity (POSSUM) physiology scores to be associated with poorer long-term survival in patients undergoing surgery for colorectal cancer (Brosens *et al*, 2006; Jenkins *et al*, 2007).

The aim of this study was to examine the relationship between patient physiology, the systemic inflammatory response and survival in patients undergoing potentially curative resection of colorectal cancer.

## Materials and methods

Patients with histologically proven colorectal cancer who, on the basis of laparotomy findings and pre-operative staging CT scan, were considered to have undergone potentially curative resection for colorectal cancer (Stages I–III) between January 1997 and December 2006 in a single surgical unit at Glasgow Royal Infirmary were included in the study. This cohort was identified from a prospectively maintained database and included both elective and emergency operations. Patients with conditions known to acutely or chronically evoke a systemic inflammatory response were excluded. These were (i) pre-operative chemo-radiotherapy, (ii) clinical evidence of infection and (iii) chronic active inflammatory disease such as active rheumatoid arthritis. Patients who died within 30 days of surgery were excluded from the survival analysis. The tumours were staged according to conventional Dukes classification (Williams *et al*, 2007).

Prospectively collected data included patient demographics, pathological characteristics of the tumour, haemotology and biochemistry results. The medical notes were then retrieved and data extracted on patient co-morbidity and physiological status. The case notes included surgical pre-operative assessment, including details of known co-morbid disease and smoking status, anaesthetic assessment, including cardiovascular evaluation and ECG interpretation, nursing notes and drug prescription charts.

Deprivation was defined using the Carstairs Deprivation Index (Carstairs and Morris, 1991). This is composed of four indicators of deprivation (car ownership, overcrowded housing, Registrar General social class and male unemployment) and has been validated for use within central Scotland (Hole and McArdle, 2002). Deprivation scores were based on the postcode of the patients' residence at the time of surgery (http://www.isdscotland.org).

The development and rationale behind the Glasgow Prognostic Score has been described previously (Forrest *et al*, 2003; McMillan, 2008). Briefly, patients with both an elevated C-reactive protein (>10 mg l^–1^) and hypoalbuminaemia (<35 g l^–1^) were allocated a score of ‘2’. Patients in whom neither of these abnormalities was present were allocated a score of ‘0’. In line with the recent modification of the Glasgow Prognostic Score, patients with an elevated C-reactive protein alone were assigned a score of ‘1’, whereas those with hypoalbuminaemia alone were assigned a score of ‘0’. All measurements of C-reactive protein and albumin were taken on admission prior to surgery.

Patient physiology was assessed by scoring patients according to the original POSSUM criteria (Copeland *et al*, 1991). Age was excluded from the physiological component of POSSUM and analysed as an independent variable, in line with previous similar studies (Tekkis *et al*, 2004; Brosens *et al*, 2006; Jenkins *et al*, 2007). The remaining 11 physiological parameters (cardiac disease, respiratory disease, ECG changes, pulse, blood pressure, haemoglobin, white cell count, sodium, potassium, urea and Glasgow Coma Scale) were used to construct a POSSUM physiology score (Table 1). Patients were then assigned to one of four groups (score 11–14, 15–20, 21–30, >30) as previously described (Tekkis *et al*, 2004).

Patients received regular follow-up (3 months, 6 months and then annually to 5 years) with CT scanning each year and regular colonoscopic surveillance until 5 years post-surgery. Information on date and cause of death was cross-checked with that received by the cancer registration system and the Registrar General (Scotland). Death records were complete until 31 August 2009, which served as the censor date for the survivors. Cancer-specific survival evaluated deaths only as a direct result of colorectal cancer in the follow-up period, whereas overall survival analysis evaluated deaths from any cause. Survival was measured from the date of surgery to the date of death.

The study was approved by the Research Ethics Committee, Glasgow Royal Infirmary, Glasgow.

### Statistics

Grouping of the variables was carried out using standard or previously published thresholds. Deaths up to September 2009 were included in the analysis. Univariate survival analysis with calculation of hazard ratios (HR) and 95% confidence intervals was carried out using the Cox proportional hazard model. A *P*-value of <0.05 was considered statistically significant. Multivariate survival analysis, using the Cox model and a stepwise backwards procedure, was carried out for all variables showing a significant association on univariate analysis. To remove a variable from the model, the corresponding *P*-value had to be >0.1. Inter-relationships between variables were assessed using contingency table analysis with the *χ*^2^ test for trend as appropriate. Analysis was performed using SPSS software (Version 15.0. SPSS Inc., Chicago, IL, USA).

## Results

Baseline clinico-pathological characteristics for all 320 patients who underwent potentially curative resection for colorectal cancer are shown in Table 2. All elective operations were carried out by one of four colorectal surgeons, whereas emergency operations were carried out by on-call general surgeons. All operations were open resections with operative technique at the discretion of individual surgeons. The majority of patients were aged 65 years or older (65%), lived in deprived areas (65%) and were current or previous smokers (58%). There was a significant association between smoking history (current or ex) and increasing deprivation (*P*=0.04). The majority of patients underwent elective operations (96%), had colonic tumours (62%), had well to moderately differentiated tumours (89%) and had node negative disease (60%). The distribution of patients by systemic inflammatory response (mGPS) and POSSUM physiology score is shown in Table 2.

**Figure 1:**
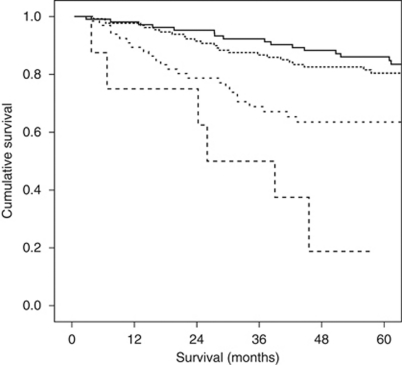
The relationship between POSSUM physiology score and cancer-specific survival in patients undergoing potentially curative resection for colorectal cancer. Groups 1–4 are shown top to bottom (*P*<0.001; log-rank test).

**Figure 2:**
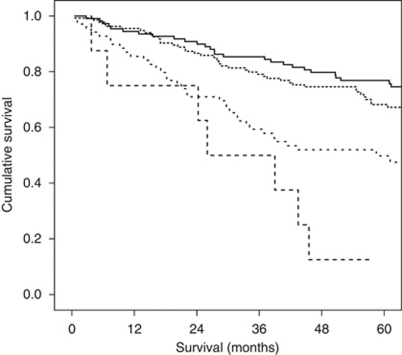
The relationship between POSSUM physiology score overall survival in patients undergoing potentially curative resection for colorectal cancer. Groups 1–4 are shown top to bottom (*P*<0.001; log-rank test).

The minimum follow-up was 32 months; the median follow-up of the survivors was 74 months. During this period, 83 patients died of colorectal cancer and there were 53 non-cancer-related deaths. The relationship between clinico-pathological characteristics and cancer-specific survival is shown in Table 2. On univariate analysis, age (*P*=0.001), smoking history (*P*=0.04), presentation (*P*<0.001), Dukes stage (*P*<0.001), mGPS (*P*<0.001) and POSSUM physiology score (*P*<0.001) were significantly associated with cancer-specific survival. The relationship between clinico-pathological characteristics and overall survival is also shown in Table 2. On univariate analysis, age (*P*<0.001), smoking history (*P*=0.004), presentation (*P*=0.001), Dukes stage (*P*=0.004), mGPS (*P*<0.001) and POSSUM physiology score (*P*<0.001) were significantly associated with overall survival. The Kaplan–Meier survival curves showing the relationship between POSSUM physiology score and both cancer specific (*P*<0.001; log-rank test) and overall survival (*P*<0.001; log-rank test) are shown in Figures 1 and 2, respectively.

On multivariate analysis, age (HR: 1.46, *P*<0.001), emergency presentation (HR: 2.08, *P*=0.08), Dukes stage (HR: 2.39, *P*<0.001), mGPS (HR: 1.78, *P*<0.001) and POSSUM physiology score (HR: 1.38, *P*=0.02) were independently associated with cancer-specific survival, whereas age (HR: 1.64, *P*<0.001), smoking history (HR: 1.52, *P*=0.02), Dukes stage (HR: 1.64, *P*<0.001), mGPS (HR: 1.60, *P*<0.001) and POSSUM physiology score (HR: 1.27, *P*=0.03) were independently associated with overall survival (Table 3).

In the group of patients with Dukes C disease, we noted a significant association between POSSUM physiology score and the likelihood of adjuvant therapy being administered (*χ*^2^=9.94, df=3, *P*=0.019). Of the 129 patients with Dukes C disease, 46 patients (36%) received adjuvant therapy and 83 patients (64%) did not. In patients with physiology score 11–14, 21 patients (51%) received adjuvant therapy; physiology score 15–20, 19 patients (35%) received adjuvant therapy; physiology score 21–30, 6 patients (21%) received adjuvant therapy; physiology score >30, no patient received adjuvant therapy. There was no significant association between the systemic inflammatory response and the likelihood of adjuvant therapy being administered in patients with Dukes C disease (*χ*^2^=4.73, df=2, *P*=0.094).

The relationships between POSSUM physiology score and clinico-pathological characteristics are shown in Table 4. The POSSUM physiology score was significantly related to all its component variables except potassium level (*P*=0.11) and Glasgow Coma Scale, the latter of which was uniformly normal. The individual physiological variables that contributed most to elevated POSSUM physiology score were haemoglobin level (abnormal in 202 out of 320), systolic blood pressure (abnormal in 192 out of 320) and cardiac function (impaired in 166 out of 320). Those that contributed least were sodium level (abnormal in 25 out of 320), potassium level (abnormal in 21 out of 320) and GCS (abnormal in 0 out of 320). A higher POSSUM physiology score was also significantly associated with increasing age (*P*<0.001), tumours of colonic origin (*P*<0.001) and advanced Dukes stage (*P*<0.05) (Table 4).

**Figure 3:**
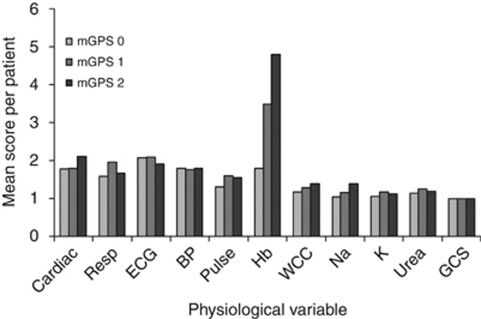
Graphic representation showing the relationship between mGPS and individual POSSUM physiological variables.

A significant relationship between POSSUM physiology score and mGPS was established (*P*=0.006) (Table 4). This relationship was further examined by calculating the mean score for each of the POSSUM component variables in patients within the mGPS groups (Figure 3). The mGPS was significantly associated with the individual physiological components of abnormal pulse rate (*P*=0.008), raised white cell count (*P*=0.05), low sodium (*P*<0.001), raised potassium (*P*=0.01) and low haemoglobin (<0.001).

## Discussion

The results of this study show that pre-operative measures of impaired patient physiology, such as elevated POSSUM physiology scores, are significantly associated with poorer cancer specific and overall survival in patients undergoing potentially curative resection of colorectal cancer. However, when considered with age, Dukes stage, smoking status and the systemic inflammatory response (mGPS), the POSSUM physiology score was reduced in statistical significance. Although the POSSUM physiology score was strongly associated with mGPS, multivariate survival analysis showed that both were independent predictors, suggesting that poor patient physiology alone cannot fully explain the relationship between the pre-operative systemic inflammatory response and reduced colorectal cancer survival.

The results of this study are consistent with previous work. Jenkins *et al* (2007) reported that, using the same thresholds, there was a significant association between an elevated POSSUM physiology score and poorer cancer-specific survival in a cohort of 432 patients with colorectal cancer. In addition, Brosens *et al* (2006) reported that, in 542 colorectal cancer patients, there was a similar association between POSSUM physiology score and 5 year overall survival using low- and high-risk groups based on the median physiology score.

Given that the POSSUM score was developed to predict post-operative mortality and morbidity, the basis of this relationship with long-term survival is not clear. One possible explanation is that poor patient physiology is associated with an increased likelihood of post-operative complications such as an anastomotic leak; recognised to be associated with early recurrence and cancer death, independent of tumour stage (McArdle *et al*, 2005; Jung *et al*, 2008; Marra *et al*, 2009). Another possible explanation, examined in this study, is that a pre-operative systemic inflammatory response reflects, in part, the existence of physiological dysfunction (Roxburgh and McMillan, 2010b). It is of interest, therefore, that Moyes *et al* (2009) recently reported that, in 455 patients undergoing colorectal cancer surgery, the pre-operative mGPS was independently associated with an increased risk of developing post-operative infectious complications. It remains to be determined whether infectious complications are the basis of the relationship between poor POSSUM physiology score, elevated mGPS and poor cancer-specific survival in patients undergoing resection of colorectal cancer. Of interest, we noted that patients with deranged physiology were significantly more likely to receive adjuvant therapy for Dukes C tumours. However, the provision of adjuvant therapy was not a predictor of cancer specific or overall survival on multivariate analysis in this cohort, suggesting that the survival benefit of good physiological function is independent of its relationship with chemotherapy.

Taken together with previous work, the results of this study pose the question as to whether pre-operative optimisation of patient physiology and down-regulation of the systemic inflammatory response may be associated with improvements in long-term survival in patients undergoing surgery for colorectal cancer. With respect to patient physiology, the components that contributed most to an increase in the POSSUM physiology score were impaired cardiac function and low haemoglobin. This would suggest that targeting these parameters would be a rational first step in the pre-operative optimisation in these patients. Indeed, there is already some evidence that the use of statins may improve survival in patients with colorectal cancer, possibly by improvement in cardiovascular status (Siddiqui *et al*, 2009). The attenuation of the systemic inflammatory response and the improvement of oxygen delivery to the tissues represent other possibilities.

In this study, the individual physiological components associated with the mGPS were an elevated pulse rate, low haemoglobin and high WCC, as well as the biochemical abnormalities of low sodium and raised potassium. A possible explanation is that poor cardiac function in these patients, combined with anaemia, may lead to relative tissue hypoxia. Indeed, it is recognised that tissue hypoxia is a potent stimulator of local and systemic inflammation (Wouters, 2005; Zinkernagel *et al*, 2007). If this were to be the case, it might be expected that the systemic inflammatory response would be more closely associated with a direct measurement of cardiopulmonary reserve, that is cardiopulmonary exercise testing (Weber *et al*, 1987). Further work is needed to define such relationships.

The results from this study have further implications. The POSSUM scoring systems have already proven to be accurate in predicting post-operative mortality (Senagore *et al*, 2004; Ferjani *et al*, 2007) and morbidity (Menon and Farouk, 2002; Valenti *et al*, 2009) after colorectal cancer surgery. Clearly, a single scoring system that would allow comparative audit of post-operative mortality, highlight patients at risk of surgical complications and predict long-term cancer survival, would be advantageous. However, although it may be argued that the majority of variables are routinely recorded as part of pre-operative assessment, the POSSUM physiology score has 11 physiological components, excluding age. In contrast, the mGPS has only two components and is, therefore, easier to construct and may be less subject to interpretative error. It remains to be determined whether the POSSUM physiology score or the mGPS will be most useful in predicting both short-term and long outcome in patients undergoing surgery for colorectal cancer.

In summary, POSSUM physiology score and the systemic inflammatory response were strongly associated and both were independent predictors of cancer specific and overall survival in patients undergoing potentially curative resection of colorectal cancer.

## Figures and Tables

**Table 1:** Physiological variables used in the construction of the POSSUM physiology score

**POSSUM physiology score**	**1**	**2**	**4**	**8**
Cardiac	Normal	Cardiac drugs	Oedema warfarin	JVP cardiomegaly
Respiratory	Normal	SOB exertion Mild COPD	SOB stairs Mod COPD	SOB rest fibrosis
ECG	Normal	—	AF (60–90)	Other abnormality
Systolic BP (mm Hg)	110–130	131–170	⩾171	⩽89
		100–109	90–99	
Pulse (beats per minute)	50–80	81–100	101–120	⩾120
		40–49		⩽39
Haemoglobin (g dl^–1^)	13–16	11.5–12.9	10–11.4	⩽9.9
		16.1–17	17.1–18	⩾18.1
White cell count ( × 10^12^ per litre)	4–10	10.1–20	⩾20.1	—
		3.1–3.9	⩽3	
Sodium (mmol l^–1^)	⩾136	131–135	126–130	⩽125
Potassium (mmol l^–1^)	3.5–5	3.2–3.4	2.9–3.1	⩽2.8
		5.1–5.3	5.4–5.9	⩾6
Urea (mmol l^–1^)	⩽7.5	7.6–10	10.1–15	⩾15.1
GCS	15	12–14	9–11	⩽8

Abbreviations: AF=atrial fibrillation; BP=blood pressure; COPD=chronic obstructive pulmonary disease; GCS=glasgow coma score; JVP=jugular venous pressure; POSSUM=physiological and operative severity score for the enumeration of mortality and morbidity; SOB=shortness of breath.

Age is excluded from the physiology score and is analysed as an independent variable.

**Table 2:** The relationship between clinico-pathological variables and survival in patients undergoing potentially curative resection for colorectal cancer; univariate survival analysis

		**Cancer-specific survival**		**Overall survival**	
**Variable**	**320 (%)**	**Hazard ratio (95% CI)**	***P*-value**	**Hazard ratio (95% CI)**	***P*-value**
*Age*					
⩽64	111 (35)				
65–74	102 (32)				
⩾75	107 (33)	1.66 (1.26, 2.18)	0.001	1.80 (1.45, 2.23)	<0.001
					
*Sex*					
Male	170 (53)				
Female	150 (47)	1.30 (0.84, 2.01)	0.25	1.18 (0.84, 1.66)	0.34
					
*Deprivation*					
1–2	12 (4)				
3–5	99 (31)				
6–7	209 (65)	1.11 (0.74, 1.65)	0.46	0.98 (0.72, 1.32)	0.77
					
*Smoking*					
Never	135 (42)				
Current/previous	185 (58)	1.62 (1.02, 2.55)	0.04	1.67 (1.17, 2.39)	0.004
					
*Presentation*					
Elective	307 (96)				
Emergency	13 (4)	3.93 (1.80, 8.56)	<0.001	3.00 (1.53, 5.94)	0.001
					
*Tumour site*					
Colon	197 (62)				
Rectum	123 (38)	0.84 (0.53, 1.32)	0.45	1.09 (0.77, 1.54)	0.62
					
*Differentiation*					
Well/ moderate	286 (89)				
Poor	34 (11)	1.26 (0.63, 2.52)	0.51	1.58 (0.96, 2.59)	0.07
					
*Dukes stage*					
Dukes A	38 (12)				
Dukes B	153 (48)				
Dukes C	129 (40)	2.21 (1.51, 3.21)	<0.001	1.60 (1.21, 2.10)	0.004
					
*Adjuvant therapy*					
No	254 (79)				
Yes	66 (21)	1.00 (0.59, 1.69)	0.99	0.90 (0.59, 1.37)	0.61
					
*mGPS*					
Low (0)	194 (61)				
Intermediate (1)	90 (28)				
High (2)	36 (11)	1.71 (1.29, 2.27)	<0.001	1.60 (1.28, 2.01)	<0.001
					
*POSSUM physiology score*					
Group 1 (11–14)	109 (34)				
Group 2 (15–20)	134 (42)				
Group 3 (21–30)	69 (21)				
Group 4 (>30)	8 (3)	1.73 (1.33, 2.25)	<0.001	1.59 (1.29, 1.96)	<0.001

Abbreviation: CI=confidence interval.

**Table 3:** The relationship between clinico-pathological variables and survival in patients undergoing potentially curative resection for colorectal cancer; multivariate survival analysis

	**Cancer-specific survival**		**Overall survival**	
**Variable**	**Hazard ratio (95% CI)**	***P*-value**	**Hazard ratio (95% CI)**	***P*-value**
Age	1.46 (1.10, 1.94)	<0.001	1.64 (1.32, 2.05)	<0.001
Smoking	1.46 (0.92, 2.32)	0.10	1.52 (1.06, 2.18)	0.02
Presentation	2.08 (0.91, 4.76)	0.08	1.70 (0.84, 3.45)	0.14
Dukes	2.39 (1.59, 3.59)	<0.001	1.64 (1.22, 2.20)	<0.001
mGPS	1.78 (1.32, 2.41)	<0.001	1.60 (1.26, 2.02)	<0.001
POSSUM physiology score	1.38 (1.05, 1.82)	0.02	1.27 (1.02, 1.58)	0.03

Abbreviations: CI=confidence interval; mGPS=modified Glasgow Prognostic Score; POSSUM=physiological and operative severity score for the enumeration of mortality and morbidity.

**Table 4:** The relationships between POSSUM physiology score and clinico-pathological characteristics in patients undergoing potentially curative resection for colorectal cancer

	**Group 1**	**Group 2**	**Group 3**	**Group 4**	
	**11–14**	**15–20**	**21–30**	**>30**	
**Variable**	**(*n*=109)**	**(*n*=134)**	**(*n*=69)**	**(*n*=8)**	***P*-value**
*POSSUM variables*					
Cardiac (1/2/4/8)	82/27/0/0	66/60/8/0	6/40/20/3	0/1/5/2	<0.001
Resp. (1/2/4/8)	86/23/0/0	80/39/13/2	32/15/19/3	2/2/3/1	<0.001
ECG (1/4/8)	109/0/0	125/3/6	26/12/31	2/1/5	<0.001
SBP (1/2/4/8)	59/50/0/0	43/75/15/1	26/35/7/1	0/4/4/0	<0.001
Pulse (1/2/4/8)	83/26/0/0	80/46/8/0	37/27/5/0	4/3/1/0	0.01
Hb (1/2/4/8)	71/37/1/0	33/36/43/22	14/14/13/28	0/3/2/3	<0.001
WCC (1/2/4)	104/4/1	103/28/3	49/19/1	1/6/1	<0.001
Sodium (1/2/4/8)	107/2/0/0	121/10/3/0	62/5/2/0	5/2/1/0	0.008
Potassium (1/2/4/8)	106/3/0/0	123/7/4/0	64/4/1/0	6/1/1/0	0.11
Urea (1/2/4/8)	107/2/0/0	114/16/4/0	62/6/1/0	2/1/4/1	<0.001
GCS (1/2/4/8)	109/0/0/0	134/0/0/0	69/0/0/0	8/0/0/0	N/A
					
*Age*					
⩽64/65–74/⩾75	57/31/21	41/41/52	13/27/29	0/3/5	<0.001
					
*Sex*					
Male/female	64/45	64/70	38/31	4/4	0.38
					
*Smoking*					
Never/current or ex	54/55	55/79	25/44	1/7	0.09
					
*Presentation*					
Elective/emergency	107/2	127/7	66/3	7/1	0.34
					
*Tumour site*					
Colon/rectum	51/58	85/49	54/15	7/1	<0.001
					
*Deprivation*					
1–2/3–5/6–7	5/42/62	5/39/93	2/19/48	0/2/6	0.47
					
*Differentiation*					
Well or mod/poor	101/8	122/12	57/12	6/2	0.08
					
*Dukes stage*					
A/B/C	22/46/41	13/67/54	3/37/29	0/3/5	0.03
					
*Glasgow Prognostic Score*					
mGPS 0/1/2	80/22/7	77/44/13	34/21/14	3/3/2	0.006

Abbreviations: mGPS=modified Glasgow Prognostic Score; POSSUM=physiological and operative severity score for the enumeration of mortality and morbidity.
